# Optical Properties of Gold Nanoparticle Assemblies on a Glass Surface

**DOI:** 10.1186/s11671-017-2107-8

**Published:** 2017-05-12

**Authors:** M. O. Stetsenko, S. P. Rudenko, L. S. Maksimenko, B. K. Serdega, O. Pluchery, S. V. Snegir

**Affiliations:** 1 V.Lashkaryov Institute of Semiconductor Physics, National Academy of Sciences of Ukraine, 45, Av. Nauky, Kyiv, 03028 Ukraine; 2 Institut des Nanosciences de Paris, Sorbonne Universités, UPMC Univ Paris-06, CNRS-UMR 7588, 4 place Jussieu, Paris, France; 3 Chuiko Institute of Surface Chemistry of National Academy of Sciences of Ukraine, Gen. Naumov str.17, Kyiv, 03164 Ukraine

**Keywords:** Gold nanoparticles, Dimers, Localized surface plasmon resonance, Coupling, Modulation-polarization spectroscopy

## Abstract

The assemblies of cross-linked gold nanoparticles (AuNP) attract lot of scientific attention due to feasible perspectives of their use for development of scaled contact electrodes. Here, we developed and tested method of solid-state formation of dimers created from small AuNP (~18 nm) cross-linked with 1.9-nonadithiol (NDT) molecules. The morphology of created coating of a glass surface and its optical-polarization properties have been studied in detail by combination of scanning electron microscopy, atomic force microscopy, UV-visible spectroscopy, and modulation-polarization spectroscopy.

The modification of localized surface plasmon resonance (LSPR) of single AuNP and their assemblies were studied by measuring of the spectral characteristics of polarization difference at all stages of synthesis. The radiative and nonradiative modes of LSPR have been analyzed in detail at different angles of incidence light. This allowed establishing relation between surface morphology of the coating and its optical properties.

**Electronic supplementary material:**

The online version of this article (doi:10.1186/s11671-017-2107-8) contains supplementary material, which is available to authorized users.

## Background

Thin films of cross-linked gold nanoparticle (AuNP) have received considerable scientific attention during the past two decades. Two- and three-dimensional arrays of metallic nanoparticles allowed to create downscaled contact electrodes [1–5]; touch sensors [6, 7]; and optically [8–10], chemically [11], and mechanically controlled [12] resistors. The architecture of nanoparticle assemblies can be controlled by employing of AuNP with various size, shape [1, 13], and by modification of the interparticle spacing. This distance can be changed by selecting of appropriate cross-linking agent, for instance alkyldithiol- [2–4] or thiol-terminated molecules with specific functional properties [9, 10]. To achieve self-organization of AuNP, the DNA chains [1, 5] can be employed also.

A variation of the interparticle spacing allows to tune optical properties of entire material by controlling amplitude and position of a localized surface plasmon resonance (LSPR) of the cross-linked nanoparticles at nanoscale [7–9, 14]. Therefore, a lot of attention is paid for this issue. An understanding of relation between morphology and optical properties of AuNP assemblies can provide a fundamental basis for further development of the scaled nanoelectronic devices based on gold nanoparticles cross-linked with organic molecules with various functional properties.

However, developing and engineering of the corresponding nanostructures with reproducible architecture and preassigned optical properties is a subject of continuous efforts [15, 16]. A lot of attentions is paid to create AuNP assemblies with controllable architecture especially AuNP dimers, since they can be considered as the model of two gold nanoelectrodes linked by molecules with required functionality. Different pathways of dimer synthesis in liquid media were employed. Some of them proposes to control relative concentration of ethanol in water colloidal solution of AuNP [15, 17], while others to use various passivation agents [16, 18], multivalent thiol ligands [19, 20], and dithiol molecules [16, 21]. However, the further application of these AuNP assemblies in a molecular electronics becomes infeasible since it require integration into electronic circuits. Thus, two main challenges exist. First is to develop suitable method of solid-state synthesis which would occur directly on a solid surface of a conductive electrode. The second one is to achieve required optical properties of an electrode covered by AuNP assemblies. Therefore, the AuNP surface density, orientation toward incident light, and interparticle distance should be controlled during synthesis. In this relation, the LSPR which is monitored by UV-visible optical spectroscopy is expected to scale with the amount of AuNP and interparticle distance. When AuNP dimers are deposited on a glass slide, the transmission spectrum exhibits two distinct extinction bands: one at a wavelength of the LSPR band of single AuNP used in the assembly and the other at a greatly red-shifted wavelength due to the plasmon coupling along the interparticle axis for AuNP dimers [22–24]. The first experimental observations of dependence of the plasmon oscillation modes from interparticle spacing of a dimer and its orientation [25, 26] toward the incident light polarization are found in a good agreement with the proposed theory [27].

Here, we report about effective and facile method of formation of AuNP dimers cross-linked with 1.9-nonadithiol (NDT) which occurs directly on a solid surface. The glass surface was chosen to model a surface of an indium tin oxide (ITO) with electrical conductivity properties. Moreover, we have used commercially available glass slides to assemble AuNP which were synthesized using Turkevich method. With this, we brought to light some experimental asperities of solid-state synthesis of dimers and difficulties of their identification. With scanning electron microscopy (SEM), atomic force microscopy (AFM), UV-visible spectroscopy, and modulation-polarization spectroscopy (MPS), we provided a practical guide about how to control functionalization of a glass surface with AuNP to create their assemblies and to study the optical properties of created material. The MPS is an effective optical technique for diagnostics and characterization of the LSPR modification at nanoscale within the films of noble metals and metal-dielectric nanocomposites [28–30]. We will show that the plasmonic effects are strongly dependent on surface morphology, i.e., dispersion of single nanoparticles on the surface, their aggregation, and dimers formation. The comparison of LSPR parameters and optical-polarization properties for corresponding nanostructures will be demonstrated in the features of the spectral characteristics of the polarization difference, *ρ(λ)* and the angle of isotropic reflection *θ*_*ρ=0*_ *(λ)*, which are measured by MPS technique.

## Methods

### Sample Preparation

**Fig. 1:**
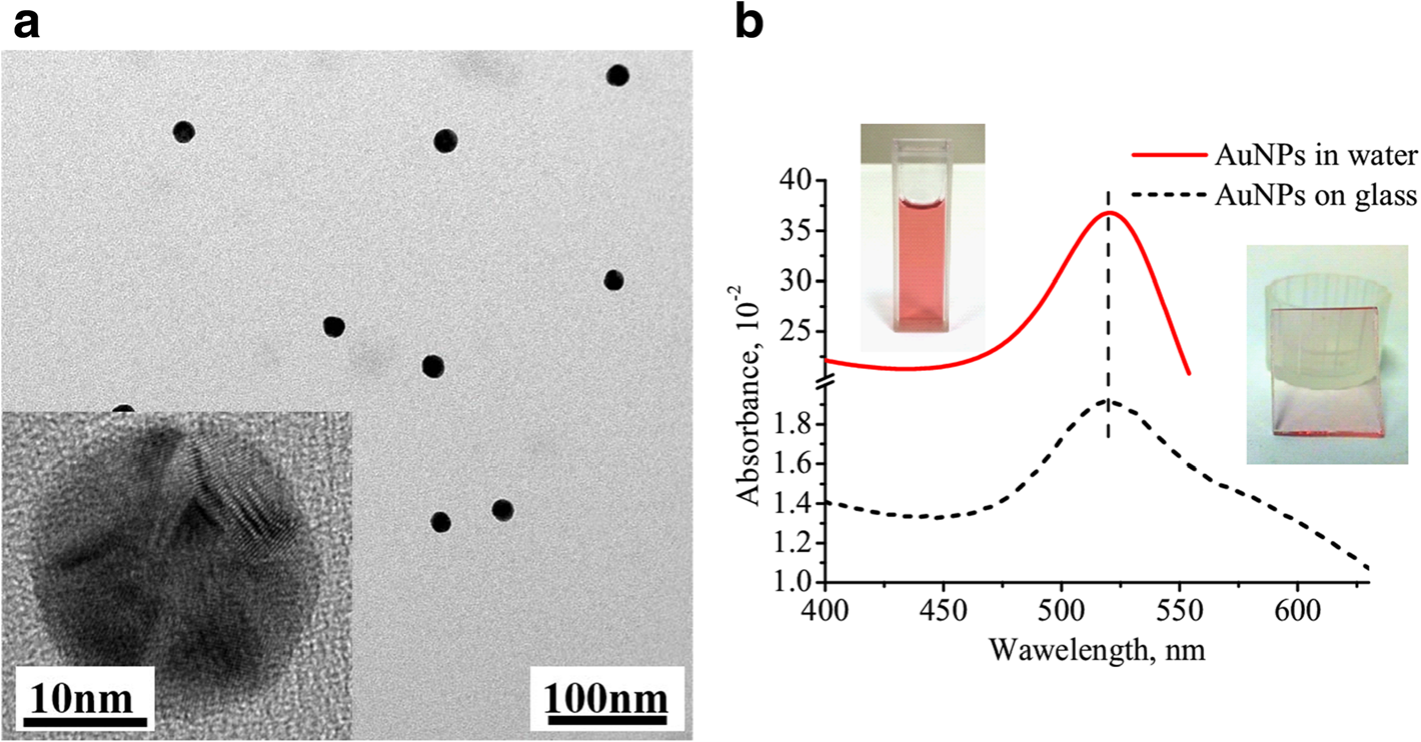
TEM image of dried colloidal solution of AuNP (**a**).The *inset* on **a** is the image of single AnNPs obtained by h-TEM with *triangular-like dark/bright* regions revealing the presence of facets on the surface AuNP. The optical spectra (**b**) of AuNP colloidal solution (*red solid curve*) and of AuNP chemically attached to a glass surface (*black dashed curve*) by means of (3-aminopropyl)-triethoxysilane (APTES) monolayer

Synthesis of AuNP occurred following the Turkevich method [31–33]. An aqueous solution of HAuCl_4_ (2.5 × 10^−4^ mol.L^−1^) was heated to the boiling point in an Erlenmeyer flask. Then 1 ml of an aqueous sodium citrate solution (1.7 × 10^−4^ mol.L^−1^) was added with vigorous magnetic stirring. The obtained colloidal water solution of gold nanoparticles was stored at 4 °C in a refrigerator to avoid nanoparticle aggregation. The average size of AuNP was ~18 nm (Fig. 1a) as evidenced by transmission electron microscopy (TEM). AuNP have a round shape with distinguishable facets on their surface which is monitored by high-resolution transmission electron microscopy (h-TEM) (Fig. 1a, insert). Optical spectra of colloidal solution (Fig. 1b) exhibit the LSPR at a wavelength of 520 nm (Fig. 1b, red curve) which is responsible for ruby color of water colloidal solution of AuNP.

**Fig. 2:**
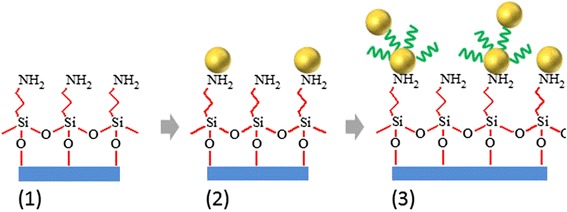
Schema of stages of AuNP dimers formation on a glass surface. **1** Glass surface functionalized by APTES. **2** First immersion into water colloidal solution of AuNP. **3** Second immersion into fresh colloidal solution of AuNP occurred after functionalization of AuNP by NDT

Glass slides (10 × 15 mm^2^) were cut from commercially available cover slips (SCHOTT). The flatness of the surface was controlled by AFM, and the average roughness did not exceed 3 nm peak to peak. These slides were carefully cleaned several times in pure ethanol and dried in a flow of dry nitrogen. Finally, they were immersed in a methanol solution of (3-aminopropyl)-triethoxysilane (APTES) (0.21 mol × L^−1^). After 3 h, the slides were sonicated in fresh methanol repeatedly (3×) to remove all physically adsorbed APTES molecules. The pre-coated glass slides (Fig. 2a, stage 1) with accessible NH_2_ groups for AuNP anchoring were subsequently immersed by one side in an aqueous solution of the AuNP (the solution should keep its color during this process). The immersion occurred using polytetrafluoroethylene tweezers. The immersing time to form the first layer of AuNPs was equal to 20 min (sample name S20) and 30 min (S30). Variation of the time allowed to control the surface density of AuNP. The obtained slides covered by AuNP (Fig. 2, stage 2) were finally rinsed several times with pure water and dried in a flow of dry nitrogen.

*Synthesis of AuNP dimers* occurred on freshly coated with AuNP glass substrates (described above). The dried slides (S20, S30) were then immersed in ethanolic solutions of NDT (*c* = 1.5 × 10^−3^ mol.L^−1^) for 30 s at ambient conditions to coat AuNP on a glass by dithiol molecule. In order to remove excessive physisorbed molecules, all substrates were rinsed successively in pure ethanol (HPLC-grade) and ultrapure water just after immersion in NDT without substrate drying. The position of initially deposited AuNP remained the same (see. Additional file 1: Figure S3). These wet substrates (S20, S30) were then immersed for the second time in a water colloidal solution of AuNP for 10 min (sample name S20/10) and 20 min (S30/20) correspondingly, with following rinsing with water and drying (stage 3). All chemicals were purchased from Sigma-Aldrich and used as received.

## Methods

The AFM characterizations of AuNP attachment was performed by a Bruker Multimode apparatus operating in the PeakForce® mode and with apparatus manufactured by NT-MDT (Russia). All measurements were performed at ambient atmospheric conditions.

Optical spectra of the films were recorded using CARY 5000 spectrometer in transmission geometry at normal incidence within the wavelength range λ = 400–700 nm. SEM of glass surface covered by AuNP was performed using Zeiss Ultra55.

Spectral polarization characteristics for AuNP assemblies and AuNP dimers were measured in Kretschmann geometry using the modulation-polarization spectroscopy technique. The scheme of setup was described in detail in [34]. The MPS technique is based on modulation of polarization state of electromagnetic radiation, when the orthogonal components of linearly polarized waves (perpendicular (*s*) and parallel (*p*)) polarizations are alternately transformed at a constant intensity, frequency, phase, and wave vector. A diffraction monochromator MDR-4 (with a halogen tube KGM-150 at the input and Franck-Ritter polarizer at the output) served as a source of spectral radiation within the wavelength range *λ* = 400–1000 nm. A photoelastic polarization modulator (PEPM) acted as a dynamic phase plate. Alternating phase incursion was caused by compression/expansion of the quartz plate. A quarter-wave mode was selected by a proper supplying voltage. As a result, linear polarization was transformed into alternating right-to-left circular polarization. A stationary quarter-wave phase plate (PP) was placed after the PEPM. By rotating PP around the optical axis, a position of PP was selected at which polarization azimuths of radiation after PP alternated between parallel and perpendicular positions relative to the incident plane (*p*- and *s*-polarization, respectively). The output radiation was directed at a photodetector PD (silicon photodiode). Reflected light was a measure of the difference of orthogonally polarized intensities, which was transformed by a PD into alternating signal. This signal was registered by a selective amplifier equipped with a phase-lock detector (lock-in-voltmeter) tuned to the modulation frequency of *f* = 50 kHz. The registered signal is the *polarization difference ρ*(*λ*,*θ*) = *r*_s_^2^–*r*_*p*_^*2*^, which is a magnitude of difference between the intensities of the internal reflection coefficients of *s*- and *p*-polarized light (*r*_*s*_^*2*^ and *r*_*p*_^*2*^, respectively). The parameter *ρ* is a *Q* component of the Stokes vector [35]. The refractive index of the quartz half-cylinder *n* = 1.456 determines the value of the critical angle of total internal reflection (TIR) as *θ*_*cr*_ = 43.6°.

When the reflection coefficients of *s*- and *p*-polarized radiation have equal amplitude values, i.e. *r*_*s*_^2^(*θ*) *= r*_*p*_^2^(*θ*), and the magnitude of polarization difference *ρ(θ)* equals zero, the light reflection occurs regardless of polarization state at the *angle of isotropic reflection θ*_*ρ=0*_ [36]. The condition of the isotropic reflection of electromagnetic radiation can be occured in the following cases: the first is a normal transmission/reflection of non-polarized radiation; the second is an attenuated internal reflection, when according to the Fresnel equations, the internal reflection coefficients of *s*- and *p*-polarized radiation are not equal at angles smaller than the critical angle (*r*_*s*_^*2*^ < *r*_*p*_^*2*^). The last case was realized in the present work. Each of these coefficients does not necessarily need to be zero. The equality of their magnitude is important.

Both *ρ* and *θ*_*ρ=0*_ parameters of MPS technique are mutually supportive and exhibit the features of spectral dependencies that caused by the optical-polarization properties of nanostructures with AuNP and characterize their resonant properties and features of surface morphology [29].

## Results

### Morphology and Optics Study

The AFM and SEM of S20 and S30 revealed that AuNP are randomly dispersed on a glass surface with some inclusion of aggregated nanoparticles (Additional file 1: Figure S1, S2). The coverage of the surface by single AuNP was calculated from AFM images and is equal to about 20/μm^2^ and 60/μm^2^ AuNP for sample S20 and S30, respectively. An analysis of the height profile (inset of Additional file 1: Figure S1b) of single AuNP confirms that they have an average diameter of ~18 nm. The color of the glass covered by AuNP becomes light pink (Fig. 1b, inset) due to LSPR of AuNP with λ_max_ = 521 nm. The color of the slides did not change with the time as well as position of AuNP during AFM measurements revealing stability of AuNP coating.

**Fig. 3:**
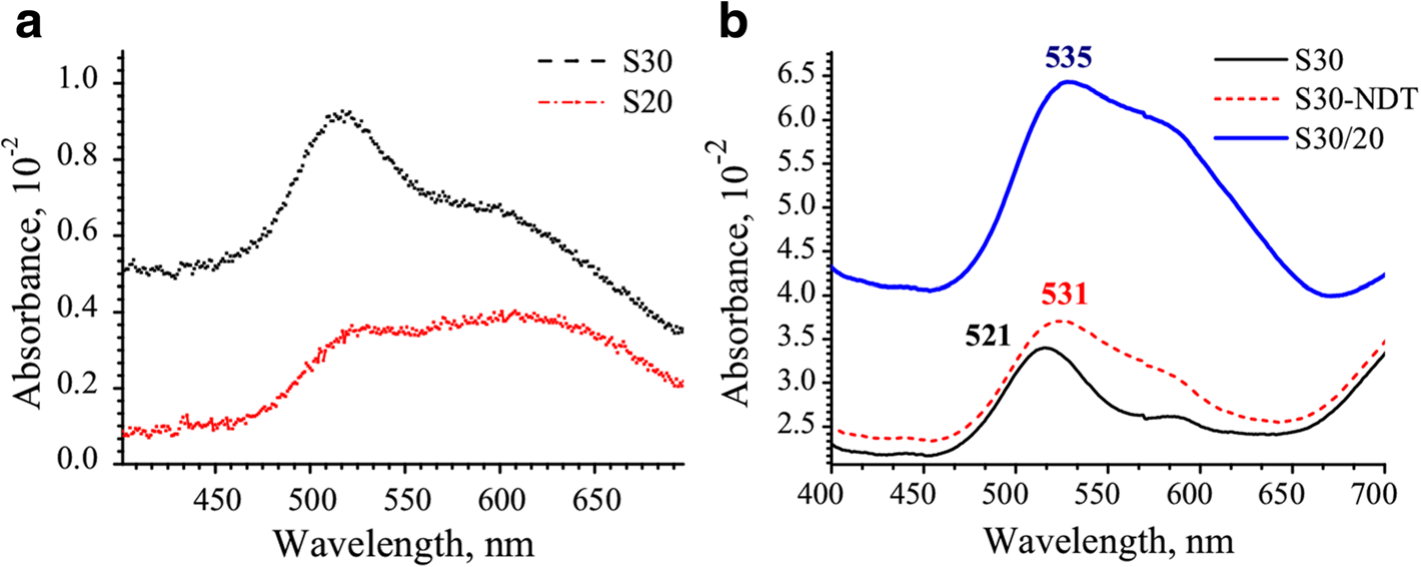
Optical spectra for S20 (*bottom curve*), S30 (*top curve*) with characteristic LSPR band of single AuNP (λ_max_ = 521 nm) (**a**) and for S30 covered by NDT with following second immersion into AuNP solution to form dimers (S30/S20) (**b**)

Among single AuNP separated by a distance larger than one diameter (~18 nm), a few nanoparticles exhibit a coupling of their LSPR. This appears in the spectrum as shoulder in the long wavelength region at 550–650 nm (Fig. 1b). These nanoparticles are arranged in the objects without any distinct shape in which AuNP are separated from each other by a distance smaller than one diameter of AuNP (Additional file 1: Figures S1, S2). Therefore, such assemblies are responsible for the formation of broad light absorption band which is shifted to the longer wavelength region compared to LSPR of single AuNP. Noteworthy, these aggregates appear first after glass immersion into colloidal solution of AuNP giving stronger light absorption band after 20 min of immersion than LSPR of single AuNP (Fig. 3a). We suspect that at initial stage of AuNP assembling on a glass, they are attracted more intensively by some locale defects of the surface. Such aggregates were systematically present on the surface in spite of multiple cleaning of the glass slides after APTES adsorption. The nature of this effect requires additional attention and studies. However, with increasing of immersion time from 20 to 30 min, the ratio of single AuNP to their aggregates is progressively rising up. This leads to increasing of the LSPR band of single AuNP (Fig. 3a).

After functionalization of the AuNP surface with NDT molecules (Fig. 2), the position of LSPR adsorption band is changed due to modification of dielectric constant of AuNP surrounding media [37, 38]. The position of LSPR band of single AuNP consequently shifts to ~10 nm to longer wavelengths (Fig. 3b). Meantime, the position of λ_max_ for AuNP aggregates did not change.

The optical spectra of the glass surface with AuNP dimers (S30/20) revealed strong increase of overall intensity (Fig. 3b). The position of λ_max_ of LSPR of single AuNP is undergoing minor changes also. The small shift to longer wavelength is attributed to appearance of longitude optical excitation mode in AuNP dimers. Similar results were observed for AuNP colloidal solution with ~20% of AuNPs dimers linked by various dithiol molecules. However, simultaneously with the formation of dimers, the number of new single AuNP attached to a glass as well as AuNP linked to existed aggregates is growing also.

**Fig. 4:**
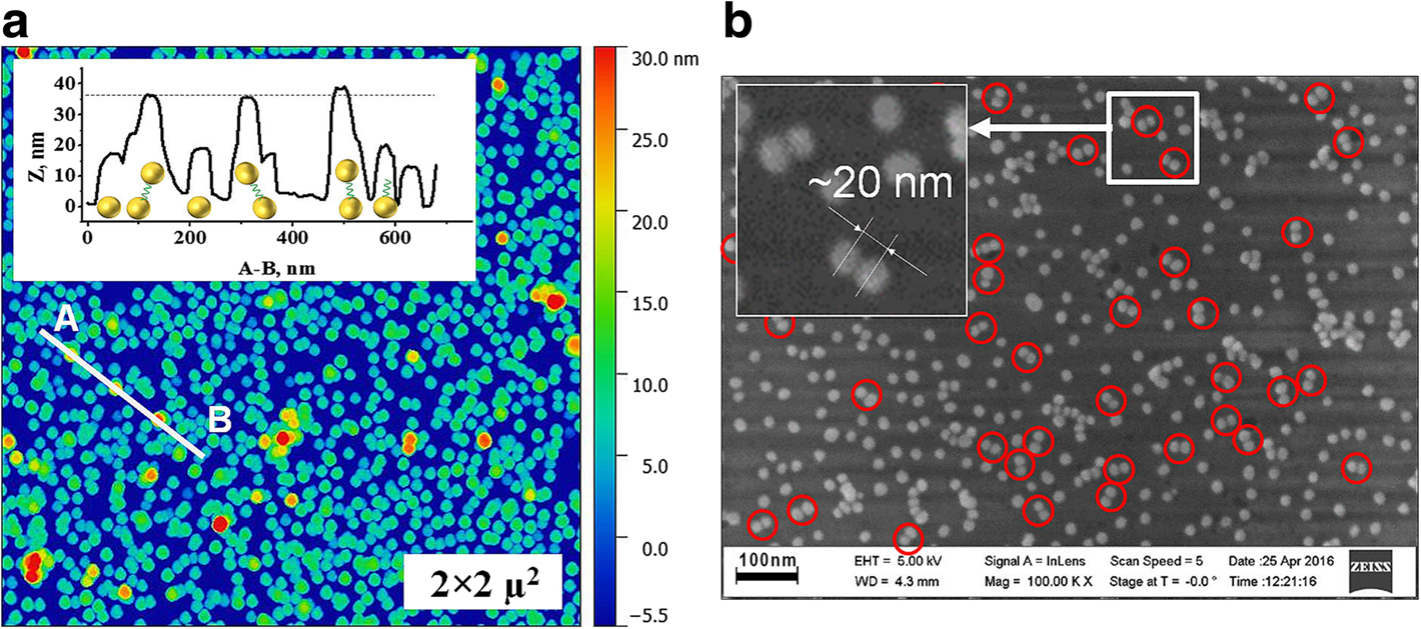
AFM (**a**) and SEM (**b**) images of the sample S30/20. The *inset* on (**a**) is the cross section along *A-B* highlighting orientation of AuNP dimers according to measured height

Thus, we performed AFM and SEM characterization of the sample S30/S20. The AFM measurements (Fig. 4a) allowed observing AuNP dimers, longitudinal axis of which is tilted to a plane of glass surface. Thus, the expected height of these dimers was in the range of 19.6–37.6 nm if considered that the length of NDT molecule and diameter of AuNP are equal to ~1.6 and 18 nm, correspondently. The quantity of such dimers, as seen from Fig. 4a, is very low. The closed-packed dimers and the ones with parallel axis to the surface were difficult to recognize due to limited resolution of AFM. This we overcame by the use of SEM (Fig. 4b). The red circles point to the positions of AuNP dimers. The measured spacing between the nanoparticles of the dimers is equal to ~20 nm. An analysis of SEM and AFM images revealed that the overall percentage of synthesized dimers is equal to about 11.6% (Table 1).

**Table 1:** The quantity of AuNP objects calculated for sample S30/20 from SEM and AFM images

	Mono	Dimers	3*x*	4*x*	5*x*	*x* > 5	Total
Quantity	259	38	12	2	8	8	327
%	79	11.6	3.7	<1	2	2	100

### MPS Study

**Fig. 5:**
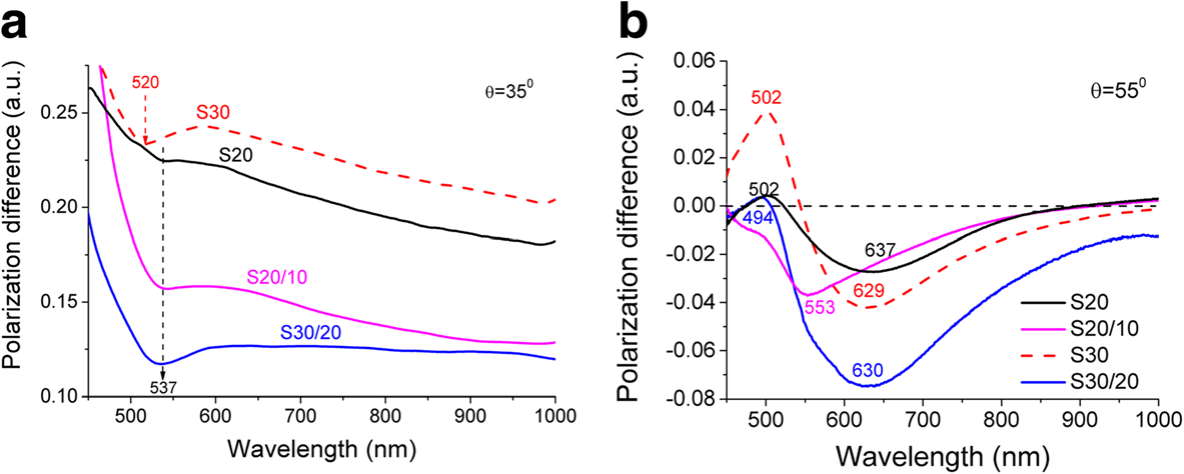
Spectral dependencies of the polarization difference *ρ*(*λ*) for samples S20, S30 of single AuNP, and S20/10, S30/20 of AuNP dimers at different incident angle of light: *θ* = 35*°* (**a**) and *θ* = 55*°* (**b**)

Optical-polarization properties and particularly plasmonic effects have been studied for the single AuNP in comparison with AuNP dimers using MPS technique by measuring the spectral characteristics of polarization difference *ρ(λ)* in the different angular regions relatively to the angle of total internal reflection *θcr* = 43.6° (Fig. 5). We have analyzed radiative (Fig. 5a) and non-radiative (Fig. 5b) modes of LSPR and paid attention to the dimers contribution in spectral characteristics *ρ(λ)*.

In Fig. 5a, the spectral characteristic of *ρ(λ)* are shown in radiative region at the incident angle of *θ* = 35° *< θcr*. All curves of *ρ(λ)* have difference in amplitude and exhibit their minima with a peak position at a wavelength of 520, 544, 542, and 535 nm for sample S30, S20, S20/10, and S30/20, respectively. This difference we analyzed further with respect to the stage of glass surface functionalization (Fig. 2) and the time of immersion of glass slides in AuNP colloidal solution.

The shortest time of the first immersion of a glass into AuNP colloidal solution was equal to 20 min (Fig. 2) and led mainly to formation of the aggregates without any distinct internal packing structure. Consequently, we observed the LSPR at 544 nm (Fig. 5a, curve S20). With the longer immersion time (S30), the number of single AuNP grew up, as it clearly indicated by UV-visible spectra on Fig. 3a. Thus, MPS revealed shift of minima from 544 nm (S20) to 520 nm (S30) (Fig. 5a) since the Frohlich frequency for small AuNP gave stronger impact on formation of the band [39, 40]. Changing of packaging density of AuNP simultaneously with formation of AuNP dimers possessing a transverse plasmon coupling mode can lead to this spectral shift [22, 23]. Among shifting, the overall amplitude value of *ρ(λ)* is increasing. Thus, the sample S30 demonstrates the highest reflection properties of single AuNP (Fig. 5a).

The correspondent growing of the amplitude values for samples S20/10 and S30/20 was also observed (Fig. 2), when second immersion occurred after covering of AuNP with NDT. However, such growing has two origins. Simultaneously with increasing of the number of single AuNP which were attached by accessible amine groups, the formation of dimers and more complex objects was observed (Fig. 4). This, consequently, led to shifting of correspondent LSPR band for S30/20 compared to S30. Interesting, the minima for the sample S30/20 has much pronounced character than S20/10. We associate this with formation of dimers on S30/20 compare to S20/10. To understand the nature of formation of this minimum (Fig. 5a, blue curve), we have studied excitation features of the LSPR in nonradiative region at the incident angle of *θ* = 55°*θ > θcr* (Fig. 5b). In the monitored wavelength region 450–1000 nm, each curve of *ρ(λ)* is characterized by the presence of two extrema. The first one lies in the short wavelength range (450–550 nm) with positive values of amplitude *ρ(λ).* It is attributed to the uncoupled dipole oscillations in single AuNP [41–43].

The second extremum is red-shifted with λ_max_ around 630 nm with negative value of amplitude of *ρ(λ).* This extremum has two origins. It may appear as the result of the coupled dipole oscillation of longitude optical excitation mode along the dimer axis between the two nanoparticles [44]. At the same time, the dipole-dipole interactions within AuNP aggregates may give additional impact on extremum formation also [45]. Both of these types of coupling create the new collective oscillation modes. These modes are lower in energy than the surface plasmon of the individual AuNP. Therefore, it gives unique opportunity to distinguish clear difference between LSPR of single nanoparticle and that one with coupled LSPR.

Firstly, we consider the spectral features of *ρ(λ)* for the functionalized glasses of AuNP with different immersion time (Fig. 5b). The reduction of AuNP quantity leads to small shift of extremum with negative values of amplitude of *ρ(λ)* to longer wavelengths from 629 to 637 nm for samples S30 and S20, respectively. If we consider that at initial stage of AuNP attaching on a glass they form aggregates (Fig. 3a), the minima for S20 at 637 nm corresponds to closely packed AuNP without any internal structure. The lateral size of these aggregates is different which explains broadening of the spectrum contour appeared as the result of overlapping of LSPR [43]. With increasing of immersion time (S20→S30), the intensity of this band displays small growing. Simultaneously with this, we have observed small shift of λ_max_ from 637 to 629 nm. We do not have a clear explanation of this minor change so far but suspect that the process of aggregation which is caused by defects on a glass surface initially (S20) may have more complex nature with the longer immersion time.

With increasing of immersion time (S20→S30), the positive value of amplitude of *ρ(λ)* at 502 nm undergo changes, as expected (Fig. 5b). This indicates that the number of single AuNP attached to the surface is growing. Initially for S20, as discussed above, only aggregates are formed on the surface. This is supported by the fact that amplitude of single AuNP at 502 nm for S20 (Fig. 5b) is much lower compare to S30.

With the formation of cross-linked AuNP using NDT alkyl dithiolthiol molecules, the intensities and what is more important shapes of all spectra (Fig. 5b) undergo changes. The shape of *ρ(λ)* is typical for nanostructure with AuNP highly ordered arrays and is caused by the domination influence of linked AuNP. This linking leads to formation of more uniform oscillation band of AuNP for S20/10 compare to disordered aggregates of S20. For sample, S20/10 the λ_max_ of long wavelength extremum of *ρ(λ)* is significantly shifted from 637 to 553 nm. This indicates about reduction of interparticle distance [39]. However, this shift cannot be attributed to significant increasing of density of single AuNP. This assumption is proved by minor changes of corresponding band at λ_max_ = 502 nm for S20/10 compare to S20. Therefore, we suspect that such progressive shift could be associated with the rearrangement of internal structure of AuNP aggregates when sample S20 was successively immersed into NDT and fresh AuNP colloidal solution. The formation of more uniformed structure of aggregates led to formation of more narrow absorption band (Fig. 5b) since the oscillation mode of cross-linked AuNP became similar (by energy) in all aggregates. We suggest following explanation of this effect. Initially, all aggregates have no distinct internal structure. As soon as aggregates became in contact with NDT solution, the surface of some AuNP in aggregates became partially modified by NDT molecules. Therefore, it is possible that due to minor mobility of these AuNP with NDT on a surface, they can become cross-linked. In addition, after the second immersion into fresh colloidal solution, these aggregates can bind even more AuNP. Consequently, the shell of aggregated object can contain AuNP separated on a distance equivalent to NDT length leading to formation of narrow light absorption band. Similar variation of the position of λ_max_ was observed in ref [46].

The behavior of the curve for sample S30/20 is different compare to S20/10. The position of the extremum of *ρ(λ)* has minor changed. An additional small reflex at λ_max_ = 553 nm might be observed. This reveals about similar rearranging of the aggregated structure like for S20/10, as discussed above. However, the amplitude value of the curve for S30/20 is essentially increased. This is caused by growing numbers of cross-linked AuNP objects (Fig. 4) including 11.6% of dimers (Table 1). The broadening of the absorption band is remained the same. Obviously, it might be expected that dimers would give a single and more pronounced reflex in the spectra compared to disordered aggregated objects since longitudinal plasmon coupling mode is sensitive to the interparticle distance [24]. However, the correspondent band is not highly pronounced since the long axis of dimers has diverse orientation toward the incident light as it shown on Fig. 4. Moreover, the longer wavelength edge of *ρ(λ)* above *λ* > 700 nm is possible associated with overlapping of plasmon’s modes of oligomers and dimers [25, 26]. This range changed only for sample S30/20 whereas for sample S20/10, it remained the same as for S20 where formation of dimers was not expected. This observation is consistent with the fact that the sample S20/10 contains mainly AuNP aggregates which give the strongest impact on formation of correspondent spectra of *ρ(λ)*.

## Discussion

**Fig. 6:**
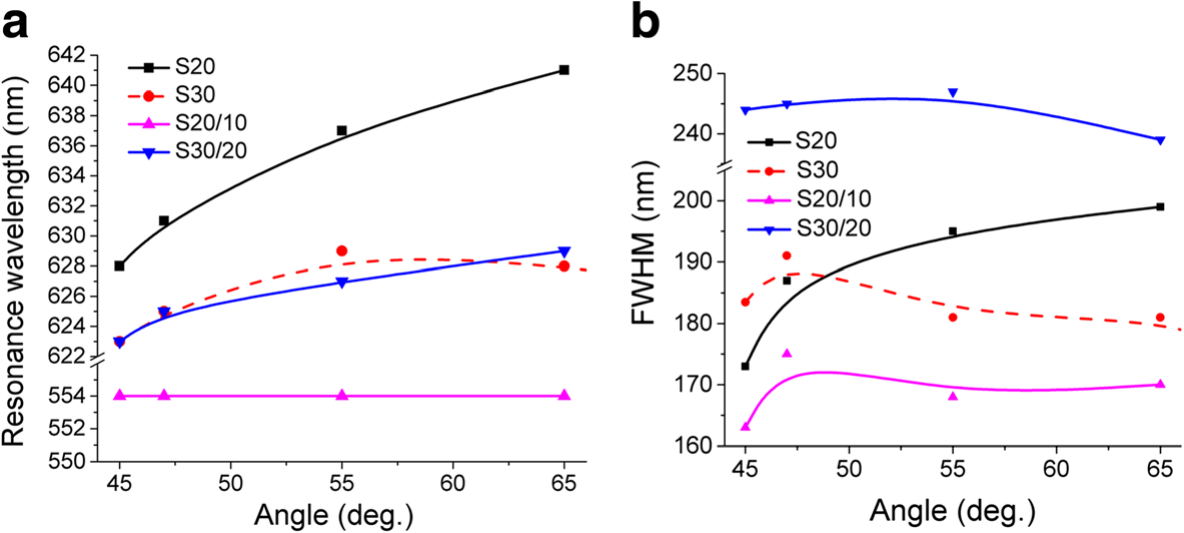
Parameters of LSPR in dependence on the incident angle *θ* for samples S20, S30, S20/10, S30/20: **a** the peak positions of resonance and **b** values of FWHM parameters

A similar experimental studies of the spectral characteristic of polarization difference were performed for different incident angles *θ* that allowed to summarize the evolution of peak positions (Fig. 6a) and values of FWHM parameters (full width at a half maximum) (Fig. 6b) of LSPR for investigated samples. An increase of incident angle *θ* leads to long-wavelength shift of LSPR and increasing of FWHM. This is observed for sample S20 due to the presence of a large number of different AuNP aggregates on a glass surface. The peak position of LSPR for S20/10 is independent on the incident angle at the reduction of corresponding values of FWHM with increasing of incident angle *θ*. In this case, the surface electromagnetic wave does not develop, and its propagation is similar to a standing wave. This is typical for nanostructure with a large number of individual AuNP and AuNP aggregates that are not interact with each other’s.

For the second type of sample S30/20 with dimers, the LSPR peak positions exhibit a weak dependency on the incident angle near a wavelength of ~ 626 nm. This result agrees with a studied of Hoon Cha and co-workers that was similarly observed a plasmon coupling in the AuNP dimers [44]. The values of FWHM parameters for dimer are increased, but on the other hand, with a weak dependency on the incident angle. It can be caused by the growing number of AuNP dimers and aggregates relatively to the single AuNP. Hence, among all investigated nanostructures, the optical-polarization features for sample S30/20 exhibited significant plasmon coupling, which shifted toward longer wavelength with increasing of the incident angle.

Note that the spectral characteristic of polarization difference includes the features of interaction between investigated nanostructures and simultaneously both parallel and orthogonal polarizations of electromagnetic radiation. They make a new contribution to the features of the spectra of *ρ(λ)* for single AuNP and their dimers. Optical properties of single AuNP and their dimers also can depend on surface morphology and expressed direction of dimer axis relative to a glass surface [22, 23].

The size of nanoparticles and interparticle spacing can lead to existence of several mechanisms of the plasmon’s interactions with electromagnetic waves on single non-interacting AuNP, their aggregates, dimers, and between them as a result of the dipole’s field interactions between adjacent AuNP [47]. Dipole plasmons of individual NPs can hybridize to form the bonding dipole plasmon mode at lower energies, giving rise to enormous electromagnetic field enhancement at the nanogap, i.e., the “hot-spot” [48, 49]. For dimer’s structure the role of gap size between nanoparticles an important and if this value is less than 2.8 nm the quantum size effect is influenced [50]. The plasmon coupling band for AuNP dimers shifts to blue wavelength region and becomes drastically broader due to disturbing of the plasmon coupling in the subnanometer regime by the electron tunneling effect [44, 51].

**Fig. 7:**
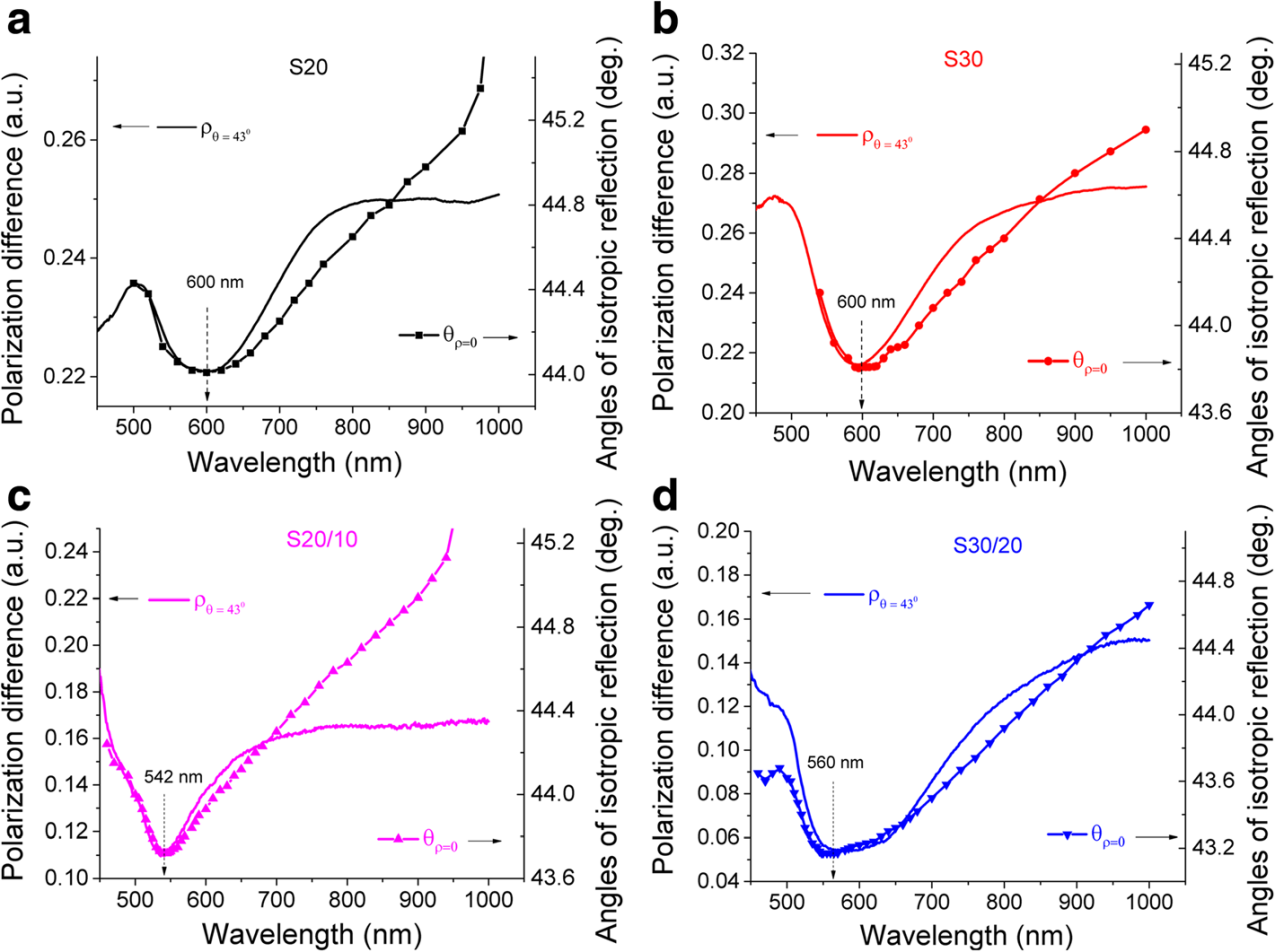
Spectral dependencies of the angles of isotropic reflection *θ*_*ρ=0*_ *(λ)* in comparison with the spectral dependencies of the polarization difference *ρ*(*λ*) at incident angle of light *θ* = 43*°* for samples S20 (**a**), S30 (**b**) of single AuNP, and S20/10 (**c**), S30/20 (**d**) of AuNP dimers

Thus, an investigation of resonant-optical properties of nanostructures with AuNP arrays depend on their sizes, shape and their mutual arrangement have been performed by measuring the spectral characteristics of the angle of isotropic reflection *θ*_*ρ=0*_ *(λ)* [29]*.* For nanostructures with single AuNP and AuNP dimers, the spectral characteristics of the angle of isotropic reflection *θ*_*ρ=0*_ *(λ)* are shown in Fig. 7 next to the appropriate spectral characteristics of polarization difference *ρ(λ)* at the incident angle of *θ =* 43°. All curves *θ*_*ρ=0*_ *(λ)* and *ρ(λ)* exhibit the resonant character with extrema that coincide at appropriate wavelength positions. The extrema of *θ*_*ρ=0*_ *(λ)* resonances at *λ* = 542 and 560 nm for samples S20/10 and S30/20, respectively, for the AuNP dimers are blue shifted relatively to λ = 600 nm for both samples S20 and S30 of single AuNP. Moreover, these bands become broader with decreasing of interparticle distance of AuNP due to formation of AuNP dimers and increase of interaction between adjacent nanoparticles into AuNP aggregates. As a result of AFM measurements (Fig. 4a), the longitudinal axis of AuNP dimers is tilted to glass surface plane (Fig. 4). Obviously, the shape of dimers and aggregates that differs from spherical can be the reason for shifting of plasmon resonances. Similar shifting of plasmon resonances in both red and blue wavelength directions in dependence on the changing of the nanoparticle shape was observed earlier [52].

In spite of the fact that the dipole-dipole interaction is attractive for *p-*polarization, which results in the reduction of the plasmon frequency (red shift of the plasmon band), while that for the *s-*polarization is repulsive, resulting in the increase in the plasmon frequency (blue shift) [41], the studying features of the isotropic reflection is unique because it reflects the change in the condition of equivalent interaction for both *s-* and *p*-polarization states of radiation that simultaneously interact with a nanostructure supported by MPS technique. It is known that natural oscillations of the conduction electrons or plasma oscillations of electrons in Au NPs are radiative modes. Correlation in extremum of the spectra of *θ*_*ρ=0*_ *(λ)* with the spectra of *ρ(λ)* is observed in the vicinity to the critical angle of TIR *θ*_*cr*_ and in closeness to the radiative region (*θ =* 43° < *θ*_*cr*_), which is caused by small mass thickness of all investigated samples and is generally associated with attenuated internal reflection of electromagnetic radiation. Apparently, the nature of the existing extrema of *θ*_*ρ=0*_ *(λ)* can be caused by average oscillations of plasmons in the AuNP due to generation of higher-order interactions between nanoparticles (quadrupole, etc.) when NP size increases and interaction transition mode from quasi-static to radiation is observed [53]. Moreover, even small intensity of exciting, electromagnetic wave can lead to strong oscillations, provided that the frequency of the incident radiation and the frequency of collective oscillations of conduction electrons in Au nanostructure are in resonance.

## Conclusions

Thus, solid-state synthesis of AuNP dimers cross-linked with NDT molecules occurred directly on a glass surface coated by amine terminated molecules. These dimers have interparticle spacing determined by the length of NDT molecule. The orientation of the dimers on a glass surface is different. Major numbers of dimers are parallel to the surface of glass plane, while others have tilted orientation. This morphology of chemically attached dimers led to broadening of UV-visible absorption spectra and appearing of features in spectra of polarization difference *ρ(λ)* under different angles of incident light. The growing number of AuNP dimers leads to broadening of LSPR band due to dominating influence of interparticle plasmon coupling that caused by decreasing of interparticle distance.

A detailed analysis of optical-polarization characteristics of LSPR in radiative and non-radiative wavelength regions allowed distinguishing between single AuNP, AuNP aggregates, and AuNP dimers in dependence on surface density of gold nanoparticles. A comparative study revealed that minor number of AuNP aggregates on a glass surface gave stronger optical absorbance compared to single AuNP. The initially disordered structure of these aggregates can undergo ordering when they are becoming in contact with NDT molecules in solution. This leads to formation of collective oscillation modes which are similar to AuNP dimers but oriented arbitrarily on the same surface. Therefore, study of the optical properties of AuNP dimers by MPS and UV-visible spectroscopy remains a complex task even when the complementary methods like AFM and SEM are used.

## Additional File

Additional file 1: Figure S1. SEM images of AuNps chemically attached to glass surface for S30 (a) and AFM image of the same surface (b) with inset for cross-section along A-B. The image was obtained using commercially available AFM (Bruker, Germany) Immesion time of a glass in AuNP colloidal solution was 30 min. Figure S2. AFM images of the glass immersed into water colloidal solution of AuNP for 20 min (S20) and for 30 min (S30). Images were obtained using commercially available AFM (NT MDT, Russia). Figure S3. AFM image of the sample S30 after immersion into NDT. (DOC 790 kb)
