# Supplementary material for: Optical Properties of Gold Nanoparticle Assemblies on a Glass Surface
Source: Nanoscale Res Lett. 2017 May 12;12:348. doi: 10.1186/s11671-017-2107-8 (PMC5427009; doi:10.1186/s11671-017-2107-8)
Supplement: Additional file 1: Figure S1. — SEM images of AuNps chemically attached to glass surface for S30 (a) and AFM image of the same surface (b) with inset for cross-section along A-B. The image was obtained using commercially available AFM (Bruker, Germany) Immesion time of a glass in AuNP colloidal solution was 30 min. Figure S2. AFM images of the glass immersed into water colloidal solution of AuNP for 20 min (S20) and for 30 min (S30). Images were obtained using commercially available AFM (NT MDT, Russia). Figure S3. AFM image of the sample S30 after immersion into NDT. (DOC 790 kb) [file 11671_2017_2107_MOESM1_ESM.doc]

Supporting information

| a)  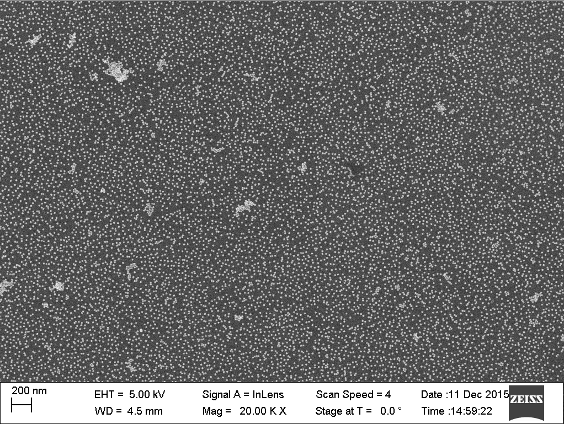 |
| --- |
| b)  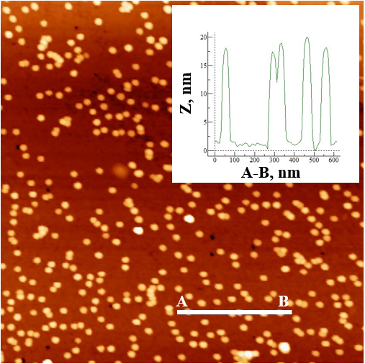  Fig 1S. SEM images of AuNps chemically attached to glass surface for S30 - (a) and AFM image of the same surface - (b) with inset for cross-section along A-B. The image was obtained using commercially  available AFM (Bruker, Germany)  Immesion time of a glass in AuNps colloidal solution  is 30 min. |

| S20  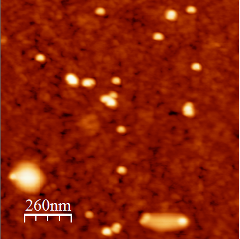 | S30  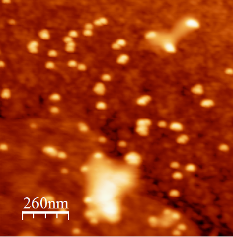 |
| --- | --- |

Figure 2S. AFM images of the glass immersed into water colloidal solution of AuNPs for 20 min (S20) and for 30 min (S30). Images were obtained using commercially  available AFM (NT MDT, Russia)


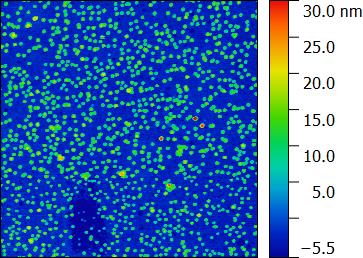


Fig.3S - AFM image the sample S30 after immersion into NDT
